# Bilateral Femoral Shaft Fractures Associated with Long-Term Bisphosphonate Therapy

**DOI:** 10.3390/diagnostics16040510

**Published:** 2026-02-08

**Authors:** Symeon Naoum, Sam Nahas, James Duncan, Charalampos Matzaroglou, Nikolaos-Achilleas Arkoudis, Maria Piagkou, Christos Koutserimpas

**Affiliations:** 1 Department of Trauma and Orthopaedics, Royal Berkshire Hospital, Reading RG1 5AN, UK; symeon.naoum@royalberkshire.nhs.uk (S.N.); sam.nahas@nhs.net (S.N.); james.duncan@royalberkshire.nhs.uk (J.D.); 2 School of Health Rehabilitation Sciences, University of Patras, 26504 Patras, Greece; matzaroglou@upatras.gr; 3 Research Unit of Radiology and Medical Imaging, National and Kapodistrian University of Athens, 11528 Athens, Greece; nick.arkoudis@gmail.com; 4 Second Department of Radiology, General University Hospital “Attikon”, National and Kapodistrian University of Athens, 12462 Athens, Greece; 5 Department of Anatomy, School of Medicine, Faculty of Health Sciences, National and Kapodistrian University of Athens, 11527 Athens, Greece; mapian@med.uoa.gr

**Keywords:** atypical femoral fracture, bisphosphonate therapy, magnetic resonance imaging, stress fracture, osteoporosis

## Abstract

Atypical femoral fractures (AFFs) are rare but increasingly recognised complications associated with prolonged bisphosphonate therapy. We present the imaging findings of a 64-year-old postmenopausal woman who developed bilateral femoral shaft stress injuries following long-term (4 years) alendronate use. Magnetic resonance imaging demonstrated Grade 3 stress injuries consistent with early incomplete atypical femoral fractures, with lateral cortical involvement and surrounding marrow oedema that preceded fracture completion. Radiographs confirmed an atypical transverse fracture pattern with lateral cortical beaking and minimal comminution. This case highlights the diagnostic value of MRI in detecting early stress-related changes prior to fracture completion, particularly in patients receiving antiresorptive therapy who present with prodromal thigh pain. Recognition of these imaging features is essential for early diagnosis, appropriate modification of osteoporosis treatment, and timely surgical intervention.

**Figure 1:**
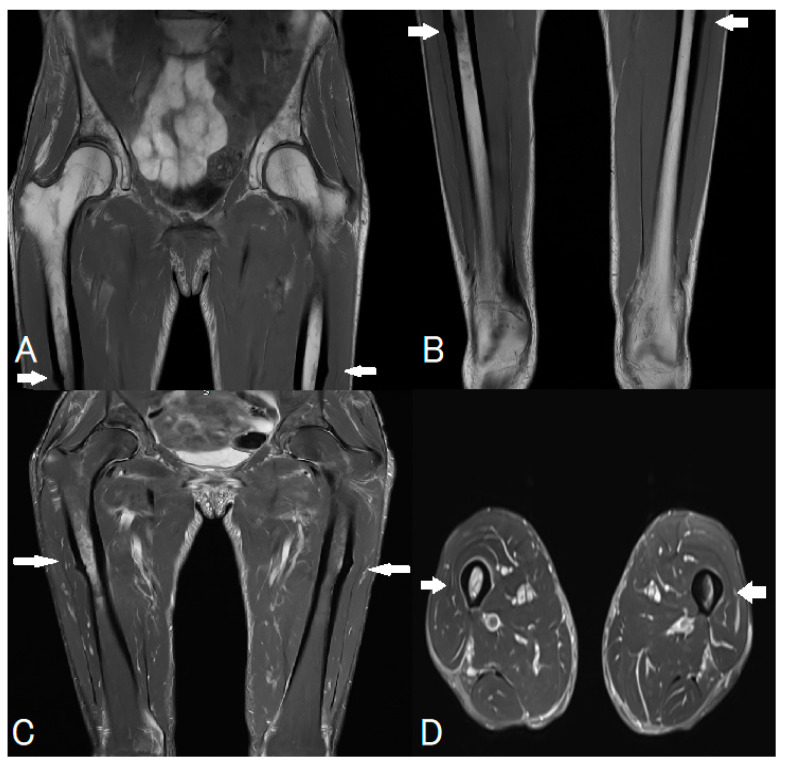
A 64-year-old postmenopausal woman with a body mass index (BMI) = 19.6 kg/m^2^ and a history of severe osteoporosis presented following a complex clinical course culminating in an acute femoral shaft fracture. Osteoporosis had been diagnosed in 2021 based on dual-energy X-ray absorptiometry (DEXA), which demonstrated markedly reduced bone mineral density (T-scores: −3.2 at the lumbar spine, −3.1 at the right hip, and −2.9 at the left hip). Both hips were assessed in accordance with international DXA recommendations to account for potential side-to-side differences and to ensure that the lowest T-score was captured for diagnostic and therapeutic decision-making [1]. Baseline serum 25-hydroxyvitamin D level was 16 ng/mL, indicating vitamin D deficiency. Oral alendronate 70 mg was initiated weekly in September 2021, together with calcium carbonate 1200 mg/day and cholecalciferol 1000 IU/day. At the time of treatment initiation, FRAX^®^ (https://www.fraxplus.org/calculation-tool, accessed on 28 January 2026)-based assessment (using femoral neck BMD) estimated a 10-year probability of major osteoporotic fracture of 11% and hip fracture of 1.6%. Follow-up DEXA after 18 months demonstrated significant improvement in bone mineral density, with T-scores of −1.8 at the lumbar spine, −2.3 at the left hip, and −2.4 at the right hip. Biochemical evaluation, including parathyroid hormone levels and screening for secondary causes of osteoporosis, was unremarkable. There was no family history of hip fracture. A history of anorexia nervosa during adolescence was noted, likely contributing to reduced peak bone mass. The patient had one full-term pregnancy at the age of 29 years. Menarche occurred at age 15 and menopause at the age of 54 years. Functionally, the patient was highly active, lived independently without walking aids, and regularly participated in running and other high-impact activities. From February 2025, she developed progressive bilateral thigh pain of insidious onset. The pain was mechanical in nature, exacerbated by weight-bearing, and gradually worsened over several months. Initially managed as a benign musculoskeletal condition, the persistence and bilateral distribution of symptoms prompted further investigation. Magnetic resonance imaging (MRI) of the pelvis, lumbar spine, and both femora (whole-femur coverage, multiplanar protocol; T1-weighted and fat-suppressed fluid-sensitive sequences) was performed to exclude referred spinal pathology and to assess for occult osseous injury. Panels (**A**,**B**) (coronal T1-weighted images) demonstrate the right and left femoral mid-diaphysis, respectively, showing focal lateral cortical thickening with a discrete linear low-signal fracture line (white arrows), consistent with developing cortical stress fractures. Panel (**C**) (coronal fat-suppressed T2-weighted image) demonstrates bilateral bone marrow oedema extending from the lateral cortex into the adjacent medullary cavity (white arrows), with associated periosteal reaction, indicating active stress response. Panel (**D**) (axial STIR image) confirms the predominant lateral cortical and periosteal involvement (white arrow) and the circumferential distribution of marrow oedema, supporting the diagnosis of atypical stress injury. No soft-tissue mass, collection, or features suggestive of infection or malignancy were identified. Overall, the imaging findings fulfilled the American Society for Bone and Mineral Research (ASBMR) 2014 criteria for atypical femoral fractures and corresponded to a Grade 3 stress injury according to the modified Fredericson–Png classification, defined by marrow oedema visible on both T1-weighted and fluid-sensitive sequences with associated cortical involvement [2,3,4,5,6,7]. These imaging findings correlated with the patient’s prodromal thigh pain and are considered highly characteristic of atypical femoral fractures, particularly in the context of prolonged bisphosphonate therapy [8,9]. Based on these MRI findings, antiresorptive therapy was discontinued, and the patient was instructed to immediately offload both lower limbs, avoid high-impact activities, and restrict exercise to low-impact activities. The imaging findings also prompted close surveillance and orthopaedic consultation for consideration of prophylactic fixation. The timeline of the case is presented in Supplementary Table S1.

**Figure 2:**
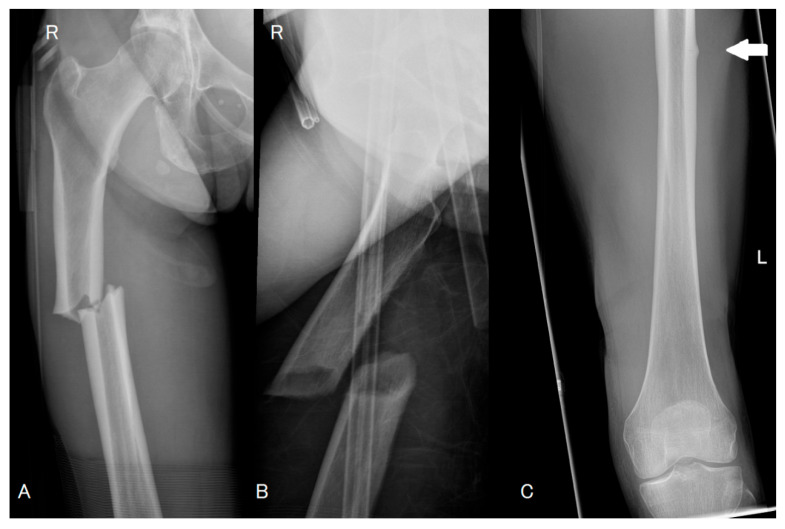
Despite these precautions, she subsequently sustained an acute injury after slipping while exiting a swimming pool and falling directly onto her right (R) side. She presented with acute right thigh pain and inability to bear weight. Plain radiographs (Panel (**A**,**B**)) demonstrated a displaced transverse fracture of the right femoral shaft with minimal comminution and characteristic lateral cortical beaking. In the context of preceding prodromal thigh pain, prior MRI evidence of bilateral stress injury, ongoing bisphosphonate therapy, and typical radiographic features, the fracture was classified as an atypical femoral fracture precipitated by low-energy trauma. She underwent surgical fixation of the right femoral fracture with intramedullary nailing [10]. Post-operatively, she progressed satisfactorily, and close surveillance of the contralateral femur was instituted in view of the well-recognised propensity for atypical femoral fractures to occur bilaterally. Following confirmation of bilateral atypical femoral fractures, ongoing bisphosphonate therapy was discontinued. Although atypical femoral fractures are most frequently associated with prolonged exposure to bisphosphonates, this case represents a recognised occurrence following a relatively shorter duration of treatment. After multidisciplinary discussion involving orthopaedic surgeons and metabolic bone specialists, anabolic therapy was initiated with teriparatide (parathyroid hormone 1–34) at a dose of 20 micrograms daily, planned for a total duration of two years, in conjunction with continued calcium and vitamin D supplementation [11,12]. Given the presence of an incomplete atypical fracture (white arrow, Panel (**C**)) in the contralateral femur (Left (L) side) and the persistent risk of progression to a complete fracture, prophylactic intramedullary nailing of the left femur was recommended and is planned in the near future [11,13]. Denosumab represents an alternative antiresorptive option in postmenopausal osteoporosis, supported by high-level evidence (Level I) for reduction in vertebral, non-vertebral, and hip fractures, and available clinical trial data indicate that it does not adversely affect fracture healing [14,15]. The long-term management strategy includes repeat dual-energy X-ray absorptiometry following completion of anabolic therapy and careful reassessment of future osteoporosis treatment, with the aim of optimising fracture prevention while minimising the risk of further atypical femoral injury. After completion of teriparatide, sequential therapy with an antiresorptive agent is planned to consolidate BMD gains and maintain anti-fracture efficacy; in the present case, depending on DXA response and residual fracture risk, transition to denosumab will be considered.

## Data Availability

Data are available upon reasonable request to the corresponding author.

## Supplementary Materials

The following supporting information can be downloaded at: https://www.mdpi.com/article/10.3390/diagnostics16040510/s1, Table S1. Timeline of osteoporosis diagnosis, investigations, and treatment.

## References

[1] Kanis J.A., Cooper C., Rizzoli R., Reginster J.Y., Scientific Advisory Board of the European Society for Clinical and Economic Aspects of Osteoporosis (ESCEO) and the Committees of Scientific Advisors and National Societies of the International Osteoporosis Foundation (IOF) (2019). European guidance for the diagnosis and management of osteoporosis in postmenopausal women. Osteoporos Int..

[2] Hwang B., Fredericson M., Chung C.B., Beaulieu C.F., Gold G.E. (2005). MRI findings of femoral diaphyseal stress injuries in athletes. Am. J. Roentgenol..

[3] Png M.A., Koh J.S., Goh S.K., Fook-Chong S., Howe T.S. (2012). Bisphosphonate-related femoral periosteal stress reactions: Scoring system based on radiographic and MRI findings. Am. J. Roentgenol..

[4] Koutserimpas C., Kotzias D., Chronopoulos E., Naoum S., Raptis K., Karamitros A., Dretakis K., Piagkou M. (2023). Suggestion of a Novel Classification Based on the Anatomical Region and Type of Bilateral Fatigue Femoral Fractures. Medicina.

[5] Fredericson M., Bergman A.G., Hoffman K.L., Dillingham M.S. (1995). Tibial stress reaction in runners. Correlation of clinical symptoms and scintigraphy with a new magnetic resonance imaging grading system. Am. J. Sports Med..

[6] Shane E., Burr D., Ebeling P.R., Abrahamsen B., Adler R.A., Brown T.D., Cheung A.M., Cosman F., Curtis J.R., Dell R. (2010). Atypical subtrochanteric and diaphyseal femoral fractures: Report of a task force of the American Society for Bone and Mineral Research. J. Bone Miner. Res..

[7] Shane E., Burr D., Abrahamsen B., Adler R.A., Brown T.D., Cheung A.M., Cosman F., Curtis J.R., Dell R., Dempster D.W. (2014). Atypical subtrochanteric and diaphyseal femoral fractures: Second report of a task force of the American Society for Bone and Mineral Research. J. Bone Miner. Res..

[8] Koutserimpas C., Piagkou M., Chronopoulos E., Raptis K., Kotzias D., Naoum S., Arkoudis N.A. (2023). Bilateral Fatigue Fractures of the Femur. J. Musculoskelet. Neuronal Interact..

[9] Yano Y., Kuriyama A., Yano Y., Takeshita A., Hashizume H. (2020). Atypical femoral fracture with bisphosphonate use. Qjm: Int. J. Med..

[10] Yoon Y.C., Oh C.W., Oh J.K., Kim J.W., Park K.H., Song H.K. (2022). Incomplete Diaphyseal Atypical Femoral Fracture due to Increased Anterolateral Bowing: Treatment with Corrective Osteotomy and Intramedullary Nailing with Augmented Plate Fixation. J. Bone Jt. Surg..

[11] Ebrahimpour A., Sadighi M., Hoveidaei A.H., Chehrassan M., Minaei R., Vahedi H., Mortazavi S.J. (2021). Surgical Treatment for Bisphosphonate-related Atypical Femoral Fracture: A Systematic Review. Arch. Bone Jt. Surg..

[12] Bégin M.J., Audet M.C., Chevalley T., Portela M., Padlina I., Hannouche D., Ing Lorenzini K., Meier R., Peter R., Uebelhart B. (2020). Fracture Risk Following an Atypical Femoral Fracture. J. Bone Miner. Res..

[13] Silverman S., Kupperman E., Bukata S. (2018). Bisphosphonate-related atypical femoral fracture: Managing a rare but serious complication. Clevel. Clin. J. Med..

[14] Gregson C.L., Armstrong D.J., Avgerinou C., Bowden J., Cooper C., Douglas L., Edwards J., Gittoes N.J.L., Harvey N.C., Kanis J.A. (2025). The 2024 UK clinical guideline for the prevention and treatment of osteoporosis. Arch. Osteoporos..

[15] Badour S., McCoy R.G., Takagi M., Everhart A.O., Parimi J., Herrin J., Karaca-Mandic P., Brito J.P. (2026). Antiresorptive Consolidation After Osteoanabolic Therapy. JAMA Intern. Med..

