# Supplementary material for: Bilateral Femoral Shaft Fractures Associated with Long-Term Bisphosphonate Therapy
Source: Diagnostics (Basel). 2026 Feb 8;16(4):510. doi: 10.3390/diagnostics16040510 (PMC12939295; doi:10.3390/diagnostics16040510)
Supplement: Supplementary file 1 [file diagnostics-16-00510-s001.zip › diagnostics-4098319-supplementary.pdf]

Supplementary table

Table S1. Timeline of osteoporosis diagnosis, investigations, and treatment

**Date**	**Investigation / Intervention**	**Site / Parameter**	**Result**	**Interpretation / Notes**
April 2021	Baseline DXA (Lunar Prodigy Advance, GE Healthcare)	Lumbar spine (L1–L3)	BMD 0.780 g/cm²; T-score −3.2; Z-score −2.1	Osteoporosis (WHO criteria)
April 2021	Baseline DXA	Left total hip	BMD 0.648 g/cm²; T-score −2.9; Z-score −2.1	Osteoporosis
April 2021	Baseline DXA	Right total hip	BMD 0.686 g/cm²; T-score −3.1; Z-score −1.7	Osteoporosis
April 2021	Baseline DXA	Left femoral neck	BMD 0.714 g/cm²; T-score −2.2; Z-score −1.1	Osteopenia
April 2021	Baseline DXA	Right femoral neck	BMD 0.711 g/cm²; T-score −2.2; Z-score −1.1	Osteopenia
April 2021	Anthropometrics	Height / weight / BMI	163 cm / 52 kg / BMI 19.6 kg/m²	Low–normal BMI
April 2021	Laboratory evaluation	25-hydroxyvitamin D	16 ng/mL	Vitamin D deficiency
September 2021	Medication initiation	–	–	Alendronate 70 mg weekly started
September 2021	Supplementation	–	–	Calcium carbonate 1,200 mg/day + cholecalciferol 1,000 IU/day
2023 (18 months)	Follow-up DXA	Lumbar spine	T-score −1.8	Improvement

2023 (18 months)	Follow-up DXA	Left total hip	T-score −2.3	Osteopenia
2023 (18 months)	Follow-up DXA	Right total hip	T-score −2.4	Osteopenia
February 2025	Clinical presentation	–	–	Progressive bilateral thigh pain
2025	MRI of both femora	–	–	Bilateral incomplete atypical femoral fractures (Grade 3 stress injury)
